# Pharmacological Approaches in Managing Symptomatic Relief of Benign Prostatic Hyperplasia: A Comprehensive Review

**DOI:** 10.7759/cureus.51314

**Published:** 2023-12-30

**Authors:** Collyn O'Quin, Kathryn L White, John R Campbell, Sarah H Myers, Shilpadevi Patil, Debbie Chandler, Shahab Ahmadzadeh, Giustino Varrassi, Sahar Shekoohi, Alan D Kaye

**Affiliations:** 1 School of Medicine, Louisiana State University Health Sciences Center, Shreveport, USA; 2 Anesthesiology, Louisiana State University Health Sciences Center, Shreveport, USA; 3 Pain Medicine, Paolo Procacci Foundation, Rome, ITA

**Keywords:** lower urinary tract symptoms, pyegeum africanum, saw palmetto, tadalafil, sildenafil, terazosin, finasteride, benign prostatic hyperplasia

## Abstract

Benign Prostatic Hyperplasia (BPH) is a prevalent condition that affects aging men, leading to the development of lower urinary tract symptoms (LUTS) and potentially severe complications such as complete obstruction. The management of BPH typically involves the use of medications from different classes, including alpha-1 antagonists, 5-alpha reductase inhibitors, and anticholinergics. Combination therapy utilizing drugs from different classes can also effectively manage the BPH-LUTS complex. Recent research has revealed that phosphodiesterase 5 (PDE5) inhibitors, including Tadalafil and Sildenafil, are highly effective in treating LUTS associated with BPH. Tadalafil as a monotherapy has recently been shown to significantly improve LUTS in BPH patients. Additionally, the use of herbal remedies as a treatment option for BPH has also been widely debated. Previous research suggests that saw palmetto can reduce BPH symptoms through several proposed mechanisms, but recent trials have found inconsistencies in its efficacy. In this literature review, we conducted an extensive PubMed database search to provide current and comprehensive insights into BPH treatment options. This review comprehensively evaluates available treatments for managing BPH, highlighting the effectiveness of different classes of medications and combination therapies in managing associated symptoms. The present investigation also discusses recent research on the efficacy of PDE5 inhibitors in treating LUTS associated with BPH and the uncertain efficacy of herbal remedies. The insights provided by this study can guide healthcare professionals in making informed decisions about managing BPH, ultimately improving patient outcomes.

## Introduction and background

Benign prostatic hyperplasia (BPH) is loosely defined as the abnormal and unregulated proliferation of prostatic stromal and epithelial cells. BPH affects more than 50% of men by the age of 60 and is histologically evident in up to 90% of men by the age of 85. Clinically, BPH is one of the most common diseases in aging men and one of the most common causes of lower urinary tract symptoms (LUTS). LUTS include increased urinary frequency, nocturia, weakened urinary stream, and may eventually lead to complications of complete urinary obstruction [1,2]. The natural disease course for BPH can be initially monitored by watchful waiting in addition to lifestyle modifications. Medical therapy should be considered if the patient’s symptom burden requires it, albeit the choice of drug therapy is dependent on the type of symptoms. 5a-reductase type 2 and androgen signaling have been discovered to play key roles in the development and maintenance of prostatic tissue [2]. Currently, the primary treatment of BPH is managed medically with agents that target hormonal and other signaling pathways that regulate and maintain prostatic growth. Targeted medical therapies include selective alpha-1 antagonists, anticholinergics, phosphodiesterase 5 (PDE5) inhibitors, 5-alpha reductase inhibitors, and beta-3 agonists. Generally, medical management of men with BPH begins with a trial of tamsulosin (a selective alpha-1 antagonist) with the potential to progress to combination therapy depending on a patient’s response and symptoms. Selective alpha-1 antagonists such as tamsulosin are fast-acting, first-line BPH treatments that target the voiding and storage of LUTS safely and effectively [3]. As stated, when monotherapy fails, combination therapy with selective alpha-1 blockers and 5-alpha reductase inhibitors is considered; however, the additional risk of adverse reactions deters many patients. The treatment of BPH is often interdisciplinary. Invasive treatment should be considered if the disease course and symptoms burden are refractory to medical and combination therapy, with transurethral resection of the prostate being the gold standard of operative management [4].

Given the prevalence of the disease, the management of symptomatic BPH has significant health and economic impacts on both patients and healthcare organizations worldwide. One limitation of the development of new treatments for BPH is that we have yet to identify a basis for the evolution of prostatic hyperplasia. While the exact etiology of prostatic hyperplasia is unknown, there is available evidence that supports the role of inflammation in the pathogenesis and progression of BPH [5]. Prostatic tissue samples from men with varying degrees of BPH have been shown to contain inflammatory infiltrates, and the presence or degree of inflammation has been shown to correlate directly with prostate volume and weight [5]. In addition to chronic inflammation, growth factor, and hormone signaling pathways have complex roles in prostatic tissue homeostasis and BPH [2]. The drawback of medical therapy emphasizes the need for novel therapeutic avenues in the treatment of BPH. As personalized medicine continues to evolve, the standard of targeted therapy will likely become dependent on the ability to risk stratify patients and identify those at higher risk for the progression of BPH [2]. In this review, therefore, we discuss current medical therapies, possible combination therapies, and natural remedies in the management of BPH.

## Review

Current medical therapies

Current medical therapies such as alpha receptor blockers, 5-alpha-reductase inhibitors, anticholinergics, and other sympathomimetics have been readily used in the treatment of BPH. Additionally, PDE5 inhibitors have recently been studied as an add-on therapy or monotherapy for the treatment of BPH.

Alpha-antagonist agents

Alpha-antagonist agents have been widely used in the management of urinary tract symptoms secondary to BPH. The American Urologic Society states alpha-blockers should be offered as monotherapy to patients with moderate to severe signs and symptoms of BPH and LUTS [6]. Systematic analyses of placebo-controlled studies show that commonly used α1-blockers (doxazosin, terazosin, alfuzosin, and tamsulosin) are statistically significantly superior to placebo in improving urinary flow and reducing symptoms [6]. Active treatment is superior to placebo in terms of improving total symptom scores (IPSS) by about 30%-45%, with an additional benefit of 10%-20% above placebo. Similarly, the overall improvement in flow (Qmax) by 15%-30% is about 10 to 15 times greater than with a placebo [6].

While alpha-blockers have equivalent efficacy, the selection of alpha-blockers may vary based on patient comorbidities and adverse event profiles. Patient age, changes in blood pressure, and ejaculatory dysfunction are considered in the selection of alpha-blockers. Regarding side effect profiles, tamsulosin does not require dose titration and can be taken once daily [6]. Alfuzosin has a more pronounced effect on blood pressure and is often discontinued due to adverse vasodilatory events, especially in patients over 75 [6]. Alpha-1 antagonists were primarily manufactured for the treatment of hypertension; however, they display significant anti-obstructive effects in the ureters, bladder, and urethral sphincter due to smooth muscle relaxation. Off-label use of one alpha-blocker in particular, terazosin, includes medical expulsive therapy for distal ureteral stones, chronic prostatitis, hyperlipidemia, and alleviation of nightmares in patients with post-traumatic stress disorder.

Recently, two new alpha-blockers, alpha-1A selective silodosin, and alpha-1D selective naftopidil, have been created for the treatment of BPH. A randomized, prospective SNIPER study suggested that a pure α1A-AR selective blocker, silodosin, has advantages in improving OAB symptoms and urinary flow compared to an α1D>A-AR selective blocker, naftopidil [7]. These results suggest that alpha-1A selective blockers are more effective for voiding symptoms. Additionally, both silodosin and naftopidil have similar safety profiles compared to pre-existing alpha-blockers [8]. Therefore, silodosin is a reasonable initial choice of treatment for patients with LUTS/BPH.

Alpha-blockers are generally well tolerated; however, statistically significant adverse effects include postural hypotension, dizziness, headaches, and nasal congestion. Rare but serious adverse effects include priapism and intraoperative floppy iris syndrome. There are no absolute contraindications to terazosin use; however, relative contraindications include geriatric populations due to the risk of orthostatic hypotension and patients with heart failure. In patients with confounding cardiovascular comorbidity, tamsulosin is well tolerated and causes less symptomatic orthostatic hypotension than terazosin [6]. BPH and prostate carcinoma can cause many similar symptoms, and both diseases usually co-exist. Hence, patients thought to have BPH should be tested before starting alpha-blocker therapy to exclude carcinoma of the prostate [8]. Additionally, American Urological Association guidelines suggest that patients with LUTS should be tested with prostate-specific antigen (PSA) regardless of the suspicion of prostatic carcinoma [6].

5-alpha reductase inhibitors

5-alpha reductase inhibitors directly target hormones contributing to the disease process to both slow progression and provide symptom relief. Dihydrotestosterone (DHT) is the primary androgen associated with prostate growth and differentiation, sebaceous gland activity, male hair patterns, and balding [9]. Decreasing the amount of DHT present in prostatic tissue decreases the glandular and stromal volume and thus prostatic size. Thus, in addition to symptomatic relief, 5-reductase inhibitors can also slow the progression of the disease course. 5α-reductase, the enzyme responsible for converting testosterone to DHT, exists in 2 forms: type 1 and type 2. Finasteride selectively inhibits type 2 whereas dutasteride inhibits both forms.

Finasteride, a 5-alpha reductase inhibitor, has been indicated for the treatment of both BPH and androgenic alopecia. To determine the efficacy of 5-reductase inhibitors in the treatment of BPH, a double-blind study was conducted evaluating the effect of two doses of finasteride (1 mg and 5 mg) with a placebo, each given once daily for 12 months, in 895 men with prostatic hyperplasia [10]. Urinary symptoms, urinary flow, prostatic volume, and serum concentrations of DHT and prostate-specific antigen were determined periodically during the treatment period [10]. As compared with the men in the placebo group, the men treated with 5 mg of finasteride per day had a significant decrease in IPSS (p<0.001), an increase of 1.6 ml per second (22%, p<0.001) in the maximal urinary-flow rate, and a 19% decrease in prostatic volume (p<0.001) [10]. Men treated with 1 mg of finasteride per day did not have a significant decrease in IPSS, but had an increase of 1.4 ml per second (23%) in the maximal urinary-flow rate, and an 18% decrease in prostatic volume [10]. Thus, a treatment of 5 mg of finasteride is beneficial in men with BPH. Side effects associated with finasteride include decreased libido, ejaculatory dysfunction, gynecomastia, and orthostatic hypotension [10]. While 5-reductase inhibitors have proven to be efficacious for the treatment of BPH, finasteride is used as an alternative treatment to alpha-blockers as it takes 6-12 months for symptomatic improvement (decreased prostatic size) to be appreciable. Finasteride is contraindicated in children, pregnant women, and/or women of childbearing age as it may cause harm to the fetus.

The availability of dutasteride, the first dual (type 1/type 2) 5α-reductase inhibitor, offers the opportunity for rapid and consistent inhibition of DHT [9]. Both short- and long-term treatment with finasteride and dutasteride result in prostate volume reduction, urinary flow rate, and symptom improvement, and a risk reduction for acute urinary retention and BPH-related surgery [9]. Additionally, dutasteride and finasteride have similar safety profiles. The most common side effect is drug-related sexual adverse events presenting in the first year.

Phosphodiesterase 5 inhibitors

PDE5 inhibitors, such as tadalafil and sildenafil, have also been studied as a treatment option for BPH. PDE5 inhibitors work by preventing PDE5 from metabolizing the second messenger molecule cyclic guanosine monophosphate (cGMP) which increases the vasodilatory response of cells. PDE5 has been observed to be at work in areas such as the smooth muscle cells of the corpus cavernosum, visceral smooth muscle, skeletal muscle, and the vasculature of the bladder, prostate, and urethra. When treating ED, PDE5 inhibitors can therefore be utilized because of the effect made on the smooth muscle cells. PDE5 inhibitors cause an increase in the cGMP concentration which allows for the relaxation of muscle cells and enhanced blood flow through penile tissues, promoting erection [11]. Accordingly, the cGMP pathway has been linked to prostate smooth muscle relaxation and reduction of detrusor muscle overactivity in the bladder as well as the relaxation of vasculature in the urogenital tract allowing for increased blood flow and relief of LUTS seen in BPH patients [12]. Hence, several studies have investigated PDE5 inhibitors as treatment options for BPH in men with and without erectile dysfunction (ED) who suffer from LUTS such as urinary frequency, urinary urgency, incomplete emptying, weak urinary stream, straining, or intermittence [12]. A recent systematic review examining the efficacy of tadalafil as monotherapy for LUTS of BPH was completed in 2021. The criteria for this review included male participants diagnosed with LUTS of BPH who either received 12 weeks of monotherapy with once daily 5 mg tadalafil or treatment with an oral isodose placebo in the same manner. About 1425 relevant articles were examined and 13 studies were ultimately chosen. Within these studies, 15 trials including 9525 participants had been completed and met the inclusion criteria [11]. The outcomes measured were changes in the total International prostate system score (IPSS), IPSS storage subscore, IPSS voiding score, IPSS quality of life, adverse events, and serious adverse events. The authors demonstrated in the review that tadalafil monotherapy produced clinically meaningful total IPSS improvement, which also significantly improved the IPSS storage and voiding scores and quality of life. There was a statistically significant increase in adverse effects in the tadalafil group including events such as headache and back pain, but there was no statistically significant difference in serious adverse events between the tadalafil and placebo groups. Therefore, the results of this systematic review suggest that tadalafil oral monotherapy (5 mg once daily for 12 weeks) significantly improved LUTS and quality of life in BPH patients, providing a promising option for treatment in the future [11]. Following this, a 2022 study investigated the use of tadalafil versus tamsulosin, a selective alpha1A-adrenergic receptor antagonist, for the treatment of LUTS in BPH patients. A total of 92 patients were assigned to two groups with one group receiving 0.4 mg of tamsulosin daily and the other group receiving 5 mg of tadalafil daily. The efficacy of the drug in each group was compared using the patient's post-void residual volume (PVRU), IPSS, and sexual health inventory for men. This study found that tadalafil and tamsulosin showed statistically similar improvements in PV [13]. Overall, the recent use of PDE5 inhibitors as add-on therapy with current medical therapies or as monotherapy for BPH shows promising results for men with LUTS from BPH, especially men with ED.

Anticholinergics

Anticholinergic medications can provide symptomatic relief to patients experiencing LUTS secondary to BPH. Oxybutynin is an anticholinergic medication indicated in patients with overactive bladder or symptoms of detrusor overactivity, including urinary frequency and urgency [14]. Regarding BPH, oxybutynin exerts its effects by causing bladder smooth muscle relaxation and relieving LUTS. Common side effects include dry mouth, constipation, somnolence, blurred vision, urinary hesitation, urinary retention, and urinary urgency. Anticholinergic therapy has historically been contraindicated in patients with LUTS associated with BPH because of concerns about developing acute urinary retention [14]. Several non-randomized clinical trials were conducted to investigate the safety and efficacy of anticholinergic therapies for LUTS associated with BPH [14]. Several trials demonstrated an increase in postvoid residual with anticholinergic therapy, which was statistically significant in two trials. Despite the increase in postvoid residual, rates of acute urinary retention were low and the drugs were well tolerated. Thus, before initiating therapy anticholinergic therapy, a postvoid residual volume should be measured to rule out baseline urinary retention [14]. Additionally, patients should be advised to discontinue this medication if LUTS worsens. Absolute contraindications for use include poorly controlled narrow-angle glaucoma, gastric dysmotility, urinary retention, and complete bladder obstruction. Relative contraindications include the elderly population as there is an increased risk of adverse side effects including altered mental status.

Beta-3 agonists

Like anticholinergics, beta-3 agonists such as mirabegron have emerged as a compelling medication for providing symptomatic relief in cases of LUTS secondary to BPH. Mirabegron has demonstrated broad effectiveness and tolerability in treating overactive bladder symptoms associated with BPH by inducing relaxation in both bladder and prostatic smooth muscle, as evidenced by recent research [15,16]. In a comprehensive systematic review encompassing four randomized controlled trials, Kang et al. underscored Mirabegron's potential, reaffirming its role as a promising therapeutic option for BPH-related LUTS, particularly when conventional treatments exhibit limitations in terms of efficacy or tolerability. Notably, mirabegron exhibited improvements in bladder function and a reduction in storage symptoms in select BPH patients. It is noteworthy; however, that this systematic review reported similar rates of adverse effects compared to anticholinergics or 5-alpha reductase inhibitors, indicating the need for further research to comprehensively assess its long-term safety and efficacy [17].

Combination therapies

Monotherapy with either alpha-blockers, antimuscarinics, or 5-alpha reductase inhibitors does not provide symptomatic relief for some patients with BPH, thus treatment for these patients often involves the use of combined agents or multiple therapies. There have been several investigations aimed at evaluating the best combination. First, a combination of PDE5 inhibitors and alpha-antagonist agents has shown to be effective and well-tolerated. Specifically, vardenafil and alpha blockers ranked higher than sildenafil and tadalafil combinations. Importantly, this combination was found to be more effective at improving LUTS compared to alpha-blockers alone [18]. Several trials have been conducted to assess the use of antimuscarinics in BPH as either single-line monotherapy or combination therapy with an alpha blocker such as terazosin. Studies show that a combined therapy of tamsulosin, an alpha blocker, and oxybutynin, an antimuscarinic, is a safe and efficacious treatment option for patients who have severe urinary storage problems or have failed medical monotherapy [19]. Additionally, recent research has explored the benefits of combining tamsulosin with mirabegron, a beta-3 adrenergic receptor agonist, which has demonstrated superior outcomes in terms of reducing LUTS and improving quality of life compared to tamsulosin alone [17,20]. Thus, combination therapy has proven effective in the treatment spectrum of the BPH-LUTS complex (Table 1).

**Table 1:** Common medical therapies for BPH BPH: Benign prostatic hyperplasia; cGMP: Cyclic guanosine monophosphate; PDE5: Phosphodiesterase 5

Drug	Drug class	Mechanism of action	Side effects	Absolute contraindications
Terazosin [7]	Alpha-adrenergic receptor blocker	Competitive antagonism of alpha-1 receptors on urothelium, sphincter smooth muscle, and urinary bladder smooth muscle relaxes and relieves obstructive symptoms	Orthostatic hypotension, headache, nasal congestion, intraoperative floppy iris syndrome, priapism	No absolute contraindications. Relative contraindications in the elderly due to potential worsening of postural hypotension and/or syncope.
Finasteride [9]	5-alpha-reductase inhibitor	Competitive inhibition of types II and III 5-alpha reductase isoenzyme results in inhibition of the conversion of testosterone to dihydrotestosterone (DHT) found in hair follicles, epididymis, seminal vesicles, vas deferens, and prostate.	Loss of libido, ejaculatory dysfunction, gynecomastia, orthostatic hypotension, post-finasteride syndrome	Children, pregnant women, and/or women of childbearing age. Those taking finasteride are prohibited from donating blood until six months following discontinuation.
Oxybutynin [13]	Anticholinergic	Blocks acetylcholine effects by competitively inhibiting muscarinic 1, 2, 3 receptors, causing bladder smooth muscle relaxation.	Dry mouth, constipation, somnolence, blurred vision, urinary hesitation, urinary retention, urinary urgency	Poorly controlled narrow-angle glaucoma, gastric dysmotility, urinary retention, bladder obstruction
Tadalafil [11]	Phosphodiesterase-5 inhibitor	Inhibition of PDE5 allows for increased levels of cGMP causing an increased vasodilatory response in various areas including smooth muscle cells and the vasculature of the genitourinary system	Non-arteritic anterior ischemic optic neuropathy, hearing loss, priapism, melanoma	Patients taking nitrates or nitroglycerin: excessive preload reduction
Mirabegron [21]	Beta-3 agonist	Activation of sympathetic beta-3 receptors resulting in dose-dependent detrusor smooth muscle relaxation	Hypertension, nasopharyngitis, urinary tract infection, dry mouth, and other anticholinergic effects	Severe uncontrolled or resistant hypertension

Herbal remedies used for BPH

Saw palmetto (Serenoa repens) has several proposed mechanisms that have been long-credited to help reduce symptoms of BPH including inhibition of 5-alpha-reductase, inhibition of the formation of DHT, inhibition of conversion of testosterone to DHT, inhibition of androgen receptor binding, and antiproliferative effects [22]. However, there are inconsistencies in treatment efficacy. Previous research found that saw palmetto-derived treatments proved to be comparable to medical treatments such as Finasteride. In “Comparison of phytotherapy (Permixon) with finasteride in the treatment of benign prostate hyperplasia: a randomized international study of 1,098 patients,” [23] researchers found Permixon (hexanic extract derived from Serenoa repens) to provide comparable relief to patients. This six-month double-blind randomized trial divided and provided patients with 320 mg Permixon and 5 mg finasteride. Patient symptoms were recorded via the International Prostate Symptom Score (IPSS) as the primary evaluation [24]. The IPSS evaluates symptoms of incomplete emptying, frequency, intermittency, urgency, stream strength, nocturia, and quality of life. Results showed that both treatment options decreased the IPSS score (lower scores are associated with improved symptoms). Permixon decreased the IPSS score by 37%, and finasteride provided about a 39% decrease with a p-value of 0.17 indicating a lack of significance. Permixon improved quality of life by 38%, and finasteride provided improved quality of life by 41%, with a p-value of 0.14, which again indicates a non-significant result. When looking at effects on the prostate, Finasteride decreased prostate volume by about 18% and decreased serum PSA levels and Permixon only decreased prostate volume by 6% (p<0.001). Finasteride also decreased serum PSA levels by 41% while Permixon provided no change in serum PSA levels (p<0.001). Again, the two differed when evaluating sexual dysfunction. Patients on finasteride had increased complaints of impotence and decreased libido (p<0.001). A limitation of this study includes a patient’s initial prostate size. Per the article, in unpublished observations, it appeared that Permixon is comparable to finasteride in patients with medium and small prostate in reducing prostate size. However, Permixon has less of an inhibitory capacity on large prostates.

More recent trials have raised questions on the efficacy of saw palmetto as an effective treatment for patients. Published in *The New England Journal of Medicine* in 2006, “Saw palmetto for benign prostatic hyperplasia” [24] used a similar study design; however, the double-blind trial took place over a year and compared the use of saw palmetto extract to a placebo. About 225 men over the age of 49 were assigned to take either saw palmetto extract 160 mg twice daily or a placebo. Groups were divided following randomization with 112 to be treated with saw palmetto and 113 to be treated with placebo. Primary outcomes involved tracking patients with the American Urological Association Symptom Index Score Questionnaire (AUASI) and maximum urine flow rate. Secondary outcomes compared the prostate size, post-void bladder volume, quality of life, and adverse effects. Results from this trial indicated that there was no improvement in symptoms or objective symptoms of benign prostate hyperplasia. There were no significant differences between mean change and AUASI scores over time between the two groups (difference in mean change 0.04 point). The saw palmetto group saw a decrease in the AUASI score of 0.68, and the placebo group had a decrease of 0.72. The same results were seen when comparing urinary flow rates; there were no significant differences found between the groups. Secondary outcomes again found similar results in that there were no significant differences noted in prostate size, PVRU, and overall quality of life. Researchers also looked at serum PSA, creatinine, and testosterone levels; these values, again, did not have any statistical significance. Some methodological limitations of this study along with other studies of saw palmetto include the fact that the active ingredient, if present, in saw palmetto is not known. Since it is not known, it is difficult to determine if a given dose is sufficient enough to provide a given effect.

Taking this a step further, even more recent trials have evaluated changing the dose. A National Institutes of Health randomized, placebo-controlled double-blind multicenter trial from 2012, “Effect of increasing doses of saw palmetto on lower urinary tract symptoms: a randomized trial” [25]. Participants were first selected as men aged at least 45 years old with a urinary peak flow rate greater than or equal to 4 ml/sec. Participants also needed an AUASI score greater than or equal to 8 but less than or equal to 24. Several other standards of requirements included recent medication use for LUTS, recent diuretic use, creatinine levels, and liver function tests among others. Patients were randomized into two groups: patients were divided into saw palmetto and placebo groups. Patients received 320 mg once daily until week 24. It was then increased to 320 mg BID at week 48, and 320 mg TID was continued to week 72. Patients were followed for 72 weeks with AUASI scores collected. Secondary outcomes were also collected to include nocturia, PVRU, prostate-specific antigen, sexual function, and prostatitis symptoms. Following completion of trial results indicated that mean AUASI scores were collected. Results indicated that mean AUASI scores decreased from 14.7 to 11.7 in placebo patients and 14.4 to 12.2 in saw palmetto patients with mean averages favoring the placebo treatment. When comparing secondary outcomes, there were no effective results seen in patients with saw palmetto treatment. While this trial’s strengths include large sample size, multi-center participation, and variations in doses of saw palmetto extract, some limitations include that only one form of saw palmetto extract was utilized. Multiple forms of preparations of saw palmetto would allow for enhanced generalizability and increased reliability.

In the most recent trials following dosing was investigated with the chemical makeup and administration of saw palmetto, a recent study from 2020, “A double blind, placebo-controlled randomized comparative study on the efficacy of phytosterol-enriched and conventional saw palmetto oil in mitigating benign prostate hyperplasia and androgen deficiency” [26]. Rather than pure saw palmetto extract, participants were also exposed to b-sitosterol-enriched saw palmetto oil. Participants in this trial were aged 40-65 years old with symptomatic BPH. They were randomized into a 12-week double-blind treatment into three groups. Patients with 500 mg b-sitosterol enriched saw palmetto oil, conventional saw palmetto oil, and placebo with 33 participants in each group. The severity of BPH was organized using the IPSS scoring system discussed above, uroflowmetry, testosterone, and PSA levels. Androgen deficiency was also evaluated using the aging male systems scale, the androgen deficiency in the aging male (ADAM) questionnaire, and serum levels of free testosterone. Results indicated that there were significant outcomes that showed improvement for patients who took the b-sitosterol-enriched saw palmetto oil. When comparing the IPSS score, for patients taking the b-sitosterol enriched saw palmetto oil it decreased from 20.00±4.41 to 16.82±4.03 by the conclusion of week 12 with a p-value of <0.0001. The decreases in value for the saw palmetto group and placebo group were not statistically significant. This raises the question of whether saw palmetto itself is comparable to a placebo, but when combined with other compounds or formulas there may be improved efficacy. A limitation of the article discussed above includes the sample size. Within each subgroup (phytosterol-enriched, conventional saw palmetto, and placebo), there were only 33 participants. While IPSS scores for patients with phytosterol-enriched saw palmetto exhibited improved scores with statistical significance, a greater sample size would benefit the ultimate statistical power.

In addition to saw palmetto, there are several other herbal compounds listed on the American Urological Association website including Cucurbita pepo seed and Pyegeum africanum. Cucurbita pepo seed (pumpkin seed oil) provides several proposed mechanisms including inhibition of 5 alpha-reductase [27], reduction in DHT levels [28], inhibition of testosterone-induced hypertrophy [27], and antitumor effects [29]. Listed below in study 5, an experimental study on rats induced with prostatic hyperplasia has shown pumpkin seed oil at doses of 2 mg/100g and 4 mg/100g of body weight to reduce prostate size [27]. A limitation of this study, however, is that the subjects studied were rats. The major limitation of this study is the fact that applying results from animal studies may not directly translate to human patients. Additionally, the short duration of treatment of 20 days may not adequately be enough time to determine valid results. Finally, this study’s lack of additional comparative groups makes it difficult to fully assess the efficacy of pumpkin seed oil.

Pyegeum africanum bark's supposed mechanism of action includes inhibition of 5 alpha-reductase [30], inhibition of DHT and estrogen receptors [31], inhibition of progesterone and androgen receptors [32], and inhibition of the basal growth of prostate stromal cells stimulated by EGF, IGF-1, bFGF, TPA, and PDBu [33]. A study by Quiles et al. (2010) [34] showed that Pyegeum africanum has antiproliferative and apoptotic effects on prostate fibroblasts and myofibroblasts but not on smooth muscle cells. The American Urological Association has listed Pyegeum africanum as a helpful supplement when taken at 75-200 mg/day in divided doses. Adverse events documented taking Pyegeum africanum are minimal and most documents include gastrointestinal events such as constipation and diarrhea (Table 2) [35]. In the article, the greatest concern is limited clinical applicability secondary to the limitations presented with in vitro study use. The cell populating in a controlled environment does not replicate the complexities seen in the human body. Additionally, the stromal cells used are a simplified sample of a human prostate when compared to the actual multicellular composition of the prostate. Further, in vivo, students would be needed to provide validation of the observed results from in vitro results.

**Table 2:** Herbal remedies used for BPH IPSS: International Prostate Symptom Score; AUASI: American Urological Association Symptom Index; BPH: Benign prostatic hyperplasia; VEGF: Vascular endothelial growth factor; DHT: Dihydrotestosterone; TGFB: Transforming growth factor - beta; FGF: Fibroblast growth factor

Author (year)	Groups studied and interventions	Results and findings	Conclusions
Study 1: Carraro et al. [23]	1,908 patients were selected and divided into two groups via double-blind randomization. The first group received 320 mg of saw palmetto oil extract (Permixon) 320 mg once daily compared to the second group who received 5 mg of finasteride.	At the end of six months, both treatments provided comparable outcomes for IPSS score decrease and quality of life score increase. However, Finasteride was able to significantly decrease prostate volume and PSA levels, while Permixon had little to no effect. Patients on finasteride had increased complaints of sexual dysfunction.	According to “Comparison of phytotherapy (Permixon) with finasteride in the treatment of benign prostate hyperplasia: a randomized international study of 1,098 patients,” patients taking 320 mg Permixon and 5 mg finasteride had similar improvement in symptoms per IPSS score and quality of life.
Study 2: Bent et al. [24]	225 men aged >49 were assigned into two groups in a double-blind trial. The trial took place over twelve months. The intervention group took 160 mg twice daily, and the other group was a placebo group.	Results from this trial indicated that there was no improvement in symptoms or objective symptoms of benign prostate hyperplasia. There were no significant differences between mean change and AUASI scores over time between the two groups (difference in mean change 0.04 points). The saw palmetto group saw a decrease in the AUASI score by 0.68, and the placebo group had a decrease of 0.72.	This year-long trial proved that there were no significant differences between saw palmetto and placebo in treating BPH.
Study 3: Barry et al. [25]	Participants participated in a 27-week randomized, placebo-controlled, double-blind, multicenter trial. Participants were men aged at least 45 years old with a urinary peak flow rate greater than or equal to 4 ml/sec with an AUASI score greater than or equal to 8 but less than or equal to 24. Patients were divided into a placebo group and an intervention group. The intervention group received 320 mg daily until week 24. It was then increased to 320 mg twice daily at week 48, and 320 mg three times daily was continued to week 72.	Results indicated that mean AUASI scores decreased from 14.7 to 11.7 in placebo patients and 14.4 to 12.2 in saw palmetto patients with mean averages favoring the placebo treatment. When comparing secondary outcomes (nocturia, post-void residual volume, prostate-specific antigen, sexual function, and prostatitis symptoms), there were again no effective results seen in patients with saw palmetto treatment.	“Effect of increasing doses of saw palmetto extract on lower urinary tract symptoms: a randomized trial” indicated that even at increased dosage and dosing intervals there was still no significant evidence that saw palmetto had any improvement in treating BPH and patients with lower urinary tract symptoms.
Study 4: Sudeep et al. [26]	In this double-blind trial, participants were also exposed to b-sitosterol-enriched saw palmetto oil. Participants in this trial were aged 40-65 years old with symptomatic BPH. They were randomized into a 12-week double-blind treatment into three groups. Patients with 500 mg b-sitosterol enriched saw palmetto oil, conventional saw palmetto oil, and placebo with 33 participants in each group.	When comparing the IPSS score, for patients taking the b-sitosterol enriched saw palmetto oil it decreased from 20.00±4.41 to 16.82±4.03 by the conclusion of week 12 with a p-value of <0.0001. The decreases in value for the saw palmetto group and placebo group with not statistically significant.	“A double blind, placebo-controlled randomized comparative study on the efficacy of phytosterol-enriched and conventional saw palmetto oil in mitigating benign prostate hyperplasia and androgen deficiency” provided results that correlated in the two above studies that conventional saw palmetto and placebo provide patients with comparable results; however, when combined with phytosterol-enriched saw palmetto, there are significant improvements in outcomes.
Study 5: Gossell-Williams et al. [27]	A population of rats was collected and prostatic hyperplasia was induced with subcutaneous testosterone (0.3 mg/100g of body weight) for 20 days. The population was divided to receive pumpkin seed oil (2.0 mg/100g and 4.0 mg/100g of body weight) or corn oil (vehicle) was also given for 20 days.	On day 21, the prostates were removed and weight was calculated. Testosterone increased prostate size in the population significantly (p<0.05). Both pumpkin seed oils displayed a decrease in prostate size; however, the 4.0 mg/100g dosage led to a significantly higher effect of prostatic growth inhibition (p<0.02).	Pumpkin seed oil may provide protective effects against testosterone-induced hyperplasia of the prostate and may be beneficial in managing BPH given the statistically significant results of this study.
Study 6: Quiles et al. [34]	Primary prostate stromal cells were collected from BPH patients undergoing prostatectomy and patients without BPH undergoing cystectomy. Pyegeum africanum was introduced to both populations of cells and proliferation assays were collected after. Levels of apoptosis, transforming growth factor B1, fibroblast growth factor 2, vimentin, alpha-smooth muscle actin, and smoothelin levels were examined following the treatment with Pyegeum africanum.	Apoptosis and antiproliferation potency were increased in BPH versus non-BPH stromal cells. There was downregulation of transforming growth factor B1 in both populations. Fibroblast growth factor 2 presence increased the cell’s sensitivity to Pyegeum africanum. However, the presence of VEGF, DHT, or beta-estradiol decreased the antiproliferative activity of Pyegeum africanum.	Pyegeum africanum has antiproliferative and apoptotic effects on prostate fibroblasts and myofibroblasts but not on smooth muscle cells. The mechanisms of action include TGFB1 downregulation and inhibition of FGF2 signaling.

Limitations

Several limitations must be acknowledged in this literature review. First, despite our extensive search strategy, the inherent limitations of conducting a literature review, including the potential omission of relevant studies, cannot be eliminated. Additionally, the focus on articles available in the PubMed database up to 2022 may have excluded more recent developments in BPH management. Second, while we aimed to provide a comprehensive overview of available treatments, the heterogeneity of the studies included, especially in terms of study design and patient populations, makes direct comparisons challenging. Furthermore, the review primarily focuses on pharmacological interventions and does not extensively cover surgical or minimally invasive procedures, which are essential aspects of BPH management. Last, the majority of the discussed treatments primarily address symptom relief, and our review does not delve deeply into the long-term outcomes, including the impact on disease progression, which remains an area of ongoing research. These limitations should be considered when interpreting the findings and in guiding future research in the field of BPH management.

## Conclusions

This comprehensive review has provided a detailed examination of the current medical therapies, potential combination treatments, and natural remedies available for managing BPH and its associated LUTS. The examined treatments, including alpha receptor blockers, 5-alpha reductase inhibitors, PDE5 inhibitors, anticholinergics, beta-3 agonists, and combination therapies, offer a nuanced approach to address the multifaceted nature of BPH.

Alpha receptor blockers, such as tamsulosin, stand out as a first-line option for symptomatic BPH. Additionally, 5-alpha reductase inhibitors like finasteride target the hormonal mechanisms driving BPH progression, providing both symptomatic relief and potential disease-modifying effects. The emerging role of PDE5 inhibitors as monotherapy, especially tadalafil, showcases their potential efficacy in managing symptoms, particularly in individuals with concurrent ED. Anticholinergic medications, like oxybutynin, and sympathomimetic agents like mirabegron, have demonstrated effectiveness in relieving LUTS, but caution is warranted due to their potential adverse effects, as detailed above. Furthermore, the promising outcomes of combination therapies highlight the value of a tailored and multifaceted approach when monotherapy proves insufficient.

While this review provides valuable insights into current medical therapies, it is essential to acknowledge certain limitations, including potential omissions of relevant studies and a focus on pharmacological interventions over operative procedures. Future research should aim to address these limitations and explore long-term outcomes and advancements beyond the scope of this review.

The implications of this paper extend beyond the current landscape of BPH management. The highlighted advancements in treatment options, especially the promising role of PDE5 inhibitors, open avenues for future research and may influence clinical guidelines. The exploration of combination therapies and the nuanced considerations for selecting specific agents based on patient comorbidities contribute to the evolving understanding of personalized BPH management.

In essence, this review serves as a valuable resource for healthcare professionals, offering evidence-based guidance to enhance decision-making in BPH management. As the field continues to evolve, this comprehensive analysis provides a foundation for further exploration and refinement of therapeutic approaches, ultimately aiming to improve the overall quality of life for individuals affected by symptomatic BPH.

## References

[1] Devlin CM, Simms MS, Maitland NJ (2021). Benign prostatic hyperplasia - what do we know?. BJU Int.

[2] Bechis SK, Otsetov AG, Ge R, Olumi AF (2014). Personalized medicine for the management of benign prostatic hyperplasia. J Urol.

[3] Plochocki A, King B (2022). Medical treatment of benign prostatic hyperplasia. Urol Clin North Am.

[4] Miernik A, Gratzke C (2020). Current treatment for benign prostatic hyperplasia. Dtsch Arztebl Int.

[5] Bostanci Y, Kazzazi A, Momtahen S, Laze J, Djavan B (2013). Correlation between benign prostatic hyperplasia and inflammation. Curr Opin Urol.

[6] Chapple CR (2005). A comparison of varying alpha-blockers and other pharmacotherapy options for lower urinary tract symptoms. Rev Urol.

[7] Yang CH, Raja A (2022). Terazosin. StatPearls [Internet].

[8] Yamaguchi K, Aoki Y, Yoshikawa T, Hachiya T, Saito T, Takahashi S (2013). Silodosin versus naftopidil for the treatment of benign prostatic hyperplasia: a multicenter randomized trial. Int J Urol.

[9] Zito PM, Bistas KG, Syed K (2022). Finasteride. StatPearls [Internet].

[10] Carson C, Rittmaster R (2003614). The role of dihydrotestosterone in benign prostatic hyperplasia. Urology.

[11] Carson CC, Rosenberg M, Kissel J, Wong DG (2014). Tadalafil - a therapeutic option in the management of BPH-LUTS. Int J Clin Pract.

[12] Cui J, Cao D, Bai Y (2021). Efficacy and safety of 12-week monotherapy with once daily 5 mg tadalafil for lower urinary tract symptoms of benign prostatic hyperplasia: evidence-based analysis. Front Med (Lausanne).

[13] Gallegos PJ, Frazee LA (2008). Anticholinergic therapy for lower urinary tract symptoms associated with benign prostatic hyperplasia. Pharmacotherapy.

[14] Ahmad MS, Dar YA, Khawaja AR (2022). Comparative study of tamsulosin versus tadalafil in benign prostatic hyperplasia patients with lower urinary tract symptoms. A prospective randomized study. Urol Ann.

[15] Calmasini FB, Candido TZ, Alexandre EC (2015). The beta-3 adrenoceptor agonist, mirabegron relaxes isolated prostate from human and rabbit: new therapeutic indication?. Prostate.

[16] Mullen GR, Kaplan SA (2021). Efficacy and safety of mirabegron in men with overactive bladder symptoms and benign prostatic hyperplasia. Curr Urol Rep.

[17] Kang TW, Kim SJ, Kim MH, Jung JH (2021). Beta 3 adrenoreceptor agonist for the management of lower urinary tract symptoms in men with benign prostatic hyperplasia: a systematic review. Int Neurourol J.

[18] Qiangzhao L, Xiaofeng Z, Fenghai Z, Qiong L, Fa Z, Bohong G, Xinsheng X (2020). Efficacy and tolerability of combination therapy with alpha-blockers and phosphodiesterase-5 inhibitors compared with monotherapy for lower urinary tract symptoms. Medicine (Baltimore).

[19] Jamal A, Srinivasarao P, Dorairajan LN (2016). Comparison of the effect of tamsulosin versus combination of tamsulosin and oxybutynin in the medical management of patients with benign prostatic hyperplasia: a randomised double blind placebo controlled study. Int J Contemp Med.

[20] Su S, Lin J, Liang L, Liu L, Chen Z, Gao Y (2020). The efficacy and safety of mirabegron on overactive bladder induced by benign prostatic hyperplasia in men receiving tamsulosin therapy: a systematic review and meta-analysis. Medicine (Baltimore).

[21] Dawood O, El-Zawahry A (2023). Mirabegron. StatPearls [Internet].

[22] Csikós E, Horváth A, Ács K (2021). Treatment of benign prostatic hyperplasia by natural drugs. Molecules.

[23] Carraro JC, Raynaud JP, Koch G Comparison of phytotherapy (Permixon) with finasteride in the treatment of benign prostate hyperplasia: a randomized international study of 1,098 patients. Prostate.

[24] Bent S, Kane C, Shinohara K, Neuhaus J, Hudes ES, Goldberg H, Avins AL (2006). Saw palmetto for benign prostatic hyperplasia. N Engl J Med.

[25] Barry MJ, Meleth S, Lee JY (2011). Effect of increasing doses of saw palmetto extract on lower urinary tract symptoms: a randomized trial. JAMA.

[26] Sudeep HV, Thomas JV, Shyamprasad K (2020). A double blind, placebo-controlled randomized comparative study on the efficacy of phytosterol-enriched and conventional saw palmetto oil in mitigating benign prostate hyperplasia and androgen deficiency. BMC Urol.

[27] Gossell-Williams M, Davis A, O'Connor N (2006). Inhibition of testosterone-induced hyperplasia of the prostate of sprague-dawley rats by pumpkin seed oil. J Med Food.

[28] Tsai YS, Tong YC, Cheng JT, Lee CH, Yang FS, Lee HY (2006). Pumpkin seed oil and phytosterol-F can block testosterone/prazosin-induced prostate growth in rats. Urol Int.

[29] Jayaprakasam B, Seeram NP, Nair MG (2003). Anticancer and antiinflammatory activities of cucurbitacins from Cucurbita andreana. Cancer Lett.

[30] Kalkman C (1966). The Old World species of Prunus subg. Laurocerasus including those formerly referred to Pygeum. Blumea: Biodiversity, Evolution and Biogeography of Plants.

[31] Allkanjari O, Vitalone A (2015). What do we know about phytotherapy of benign prostatic hyperplasia?. Life Sci.

[32] Fourneau C, Hocquemiller R, Cavé A (1996). Triterpenes from Prunus africana bark. Phytochemistry.

[33] Paubert-Braquet M, Monboisse JC, Servent-Saez N (1994). Inhibition of bFGF and EGF-induced proliferation of 3T3 fibroblasts by extract of Pygeum africanum (Tadenan®). Biomed Pharmacother.

[34] Quiles MT, Arbós MA, Fraga A, de Torres IM, Reventós J, Morote J (2010). Antiproliferative and apoptotic effects of the herbal agent Pygeum africanum on cultured prostate stromal cells from patients with benign prostatic hyperplasia (BPH). Prostate.

[35] Wilt T, Ishani A, Mac Donald R, Rutks I, Stark G (2002). Pygeum africanum for benign prostatic hyperplasia. Cochrane Database Syst Rev.

